# Prevalence of timely breastfeeding initiation and associated factors in Dembecha district, North West Ethiopia: a cross-sectional study

**DOI:** 10.1186/s13006-016-0087-4

**Published:** 2016-10-06

**Authors:** Abebe Bimerew, Muluken Teshome, Getachew Mullu Kassa

**Affiliations:** 1 Save the Children International, Bahirdar, Ethiopia; 2 Public Health Department, Medicine and Health Sciences College, Debre Markos University, Debre Markos, Ethiopia; 3 Midwifery Department, Medicine and Health Sciences College, Debre Markos University, Debre Markos, Ethiopia

**Keywords:** Breastfeeding, Timely initiation of breastfeeding, Child health, Ethiopia

## Abstract

**Background:**

Early initiation of breastfeeding is a simple and cost effective intervention to advance the health of mothers and newborn babies. A large number of neonatal deaths could be prevented if infants were breastfed. However, there is poor practice related to breastfeeding initiation within the first one hour of birth, and the factors affecting it are not well understood. This study was conducted to assess the prevalence of timely breastfeeding initiation and associated factors in Dembecha district, North West Ethiopia.

**Methods:**

A cross-sectional study design was conducted from August to September 2015. Multistage sampling techniques were used to select a total of 739 mothers who had children under 2 years of age. A pretested structured questionnaire was used to collect data. Descriptive analysis, bivariate and multiple logistic regression analysis were performed.

**Results:**

The level of timely initiation of breastfeeding was 73.1 %. The magnitude of prelacteal feeding and colostrum feeding in this study was 11.9 and 76.2 % respectively. Timely initiation of breastfeeding was significantly associated with the presence of four and above antenatal appointments during the last pregnancy (Adjusted Odds Ratio [AOR] 3.1; 95 % Confidence Interval [CI] 1.2, 8.0), access to mass media such as radio or television (AOR 1.54; 95 % CI 1.10, 2.20), and mothers who were attended by traditional birth attendant during their last birth (AOR 0.23; 95 % CI 0.07, 0.75).

**Conclusions:**

The level of timely initiation of breastfeeding was relatively good compared with previous studies in Ethiopia, although more than quarter of mothers didn’t start breastfeeding within the first one hour of birth. Timely initiation of breastfeeding was significantly associated with the presence of four and above antenatal care during the last pregnancy, access to mass media (e.g. radio, television), and last child attended by traditional birth attendant. Programs should encourage mothers to use skilled birth attendants at birth, emphasise the importance of feeding colostrum and to initiate breastfeeding within one hour after childbirth.

## Background

The timely initiation of breastfeeding is defined as putting the newborn baby to the breast within one hour of birth [1]. Breastfeeding is an important public health strategy to reduce infant, child and maternal morbidity and mortality and helps to control health care costs. Breastfeeding is associated with a reduced risk of otitis media, gastroenteritis, respiratory illness, sudden infant death syndrome, necrotizing enterocolitis, obesity, and hypertension [2].

Based on World Health Organization (WHO) report, globally over one million newborn infants could be saved each year by initiating breastfeeding within the first hour of life. In developing countries alone, early initiation of breastfeeding could save as many as 1.45 million lives each year by reducing deaths mainly due to diarrheal diseases and lower respiratory tract infections in children [3]. Studies have also reported that timely initiation of breastfeeding can reduce more than twenty percent of neonatal deaths [4]. In developing countries where the rate of communicable diseases is high, timely initiation of breastfeeding is important in reducing the diarrheal disease in the child [5].

According to study conducted in India, timely initiation of breastfeeding is recognized as the first and vital step toward reducing the infant and less than 5 years of age child mortality. It has the potential to prevent 16 % of neonatal deaths if all infants are breastfed from day 1, and 22 % if breastfeeding is started within the first hour [6].

For several years, the poor nutritional status of children and women in Ethiopia has caused serious health problems [7]. Based on 2010 Federal Ministry of Health (FMOH) report, the initiation of breastfeeding within one hour of birth was lowest in the Amhara and Somali regions (38 and 40 %, respectively), and highest in the South Nation Nationality of People (SNNP) and Dire Dawa regions (67 and 66 %, respectively [8]. A study conducted in south east Ethiopia showed the prevalence of timely initiation of breastfeeding to be 52.4 %. The study also showed that factors that were associated with timely breastfeeding initiation were being urban resident and getting postnatal counselling [9]. A community based cross-sectional study conducted in western Ethiopia also showed a better, 88.5 % prevalence of timely breastfeeding initiation. Getting advice on breastfeeding during antenatal visits, women who knew the importance of colostrum and importance of mother to child attachment were the determinant factors which were associated with timely breastfeeding initiation [1].

Other studies have showed that problems related with the timely initiation of breastfeeding cause a rise in neonatal mortality of 22 % [10]. This study will be conducted to assess the prevalence of the timely initiation of breastfeeding and associated factors in Dembecha Woreda, North West Ethiopia. The findings of this study will be important for planning and implementation of prevention strategies of child morbidity and mortality in resource limited setting.

## Methods

### Study design, area and period

A cross-sectional quantitative study was conducted in Dembecha Zuria Woreda, from August to September, 2015. Dembecha District is one of the fifteen Districts in West Gojjam zone of Amhara regional state, Ethiopia. Dembecha town is the capital town of Dembecha Zuria Woreda. The woreda is located 349 km north from the capital city of Addis Ababa and 215 km south from the regional capital town of Bahir Dar. The woreda has 25 rural kebeles and 4 urban kebeles [11]. Based on 2014/ 2015 Amhara Bureau of Finance and Economic Development Report, the total population of the district was estimated to be 151,020. Of this, 49.5 % were females and 50.5 % were males. The total number of women who had a child less than 2 years of age in the woreda was 5434. The majority, 95 % of the population follow Ethiopian Orthodox Christianity. The woreda has six health centres which provide healthcare services [11].

### Source and study population

The source population were all mothers who had at least one child less than 2 years of age and were living in the woreda. Mothers who have a child less than 2 years of age, who are permanent residents, and who lived in the study area for at least six months, were included in the study.

### Sample size determination

The required sample size of the study was determined by using a single population proportion formula with the following assumptions:

n = total number of mothers to be interviewed

z = critical value at 95 % confidence interval (1.96)

p = prevalence of timely initiation of breastfeeding in under two children which is 57.2 % in rural communities of Arba Minch Zuria [12].

d = marginal error between sample statistics and the population parameter (5 %)

$$ \mathrm{n} = \frac{{\left(\mathrm{z}\frac{\upalpha}{2}\right)}^2\mathrm{p}\ \left(1-\mathrm{p}\right)}{{\mathrm{d}}^2} $$

$$ \mathrm{n} = \frac{1.96^2*0.572*0.428}{0.05^2} = 376 $$

Since the source population was 5434, less than 10,000, the correction formula was used and gave the sample size of 352, but since multistage sampling technique was used, the sample size is multiplied by the design effect to get the final sample size. By taking the design effect as two, the required sample size was 704. Additionally 5 % was added for non-response rate giving a final sample size of 739.

### Sampling procedure

**Fig. 1:**
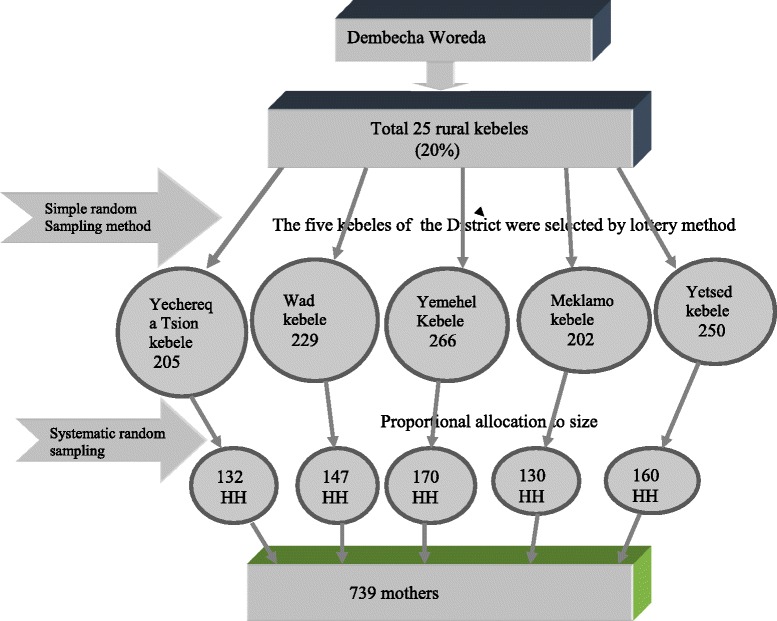
Schematic frame work of the sampling procedure

Multistage sampling technique was used to select the study population at the community level. The first 5 kebeles were selected from 25 kebele using simple random sampling technique. A sample size (739) of mothers who had a child less than 2 years old from each kebele was selected proportionally and each household was selected by systematic random sampling technique. Then, the number of house hold (HH) with mothers and a child less than 2 years of age were selected from the sampled kebeles using the family folder registration book of each house hold, found in the health post. Based on the proportional allocation each sample was selected every unit of second household. In the case when more than one mother with in a household were available, a lottery method was used to select the mother to be included and if more than two under two year children were found in the house hold the youngest child was selected. The sampling procedure of children under 2 years of age in Dembecha Zuria Woreda is presented in Fig. 1.

### Variables of the study

The dependent variable of this study is the timely initiation of breastfeeding, which means the initiation of breastfeeding within one hour after childbirth. The independent variables include; sociodemographic characteristics (age, religion, education of the mother, education of the husband, ethnicity, marital status, economic status/monthly income, sex of infant, age of infant, occupation of the mother, occupation of the husband and access to Media). Obstetric related history (parity, history and frequency of antenatal visits, counselling, place of delivery, mode of delivery, postnatal follow up, history of breast problems, and knowledge and attitude of the mother towards breastfeeding.

### Operational definitions

#### Timely initiation of breastfeeding

Is defined as putting the neonate on the mother’s breast to suckle within one hour of birth as reported by the mother/caretaker of the child.

#### Knowledge

Iincluded breastfeeding initiation, and an awareness and understanding of the timely initiation of breastfeeding. Mothers were considered to have good knowledge if they correctly answered greater than or equal to 70 % of the total knowledge related assessing questions.

#### Attitude

In this study is defined as the mothers overall favourableness toward the timely initiation of breastfeeding. Respondents were categorized as having a favourable attitude if the final score for attitude related questions was greater than or equal to 50 %. If respondents had a score of less than 50 %, they were grouped as having an unfavourable attitude towards the timely breastfeeding initiation.

#### Traditional birth attendant

Is defined when the childbirth was attended by an unskilled personnel such as relatives, neighbour, friend or unskilled community based birth attendants.

### Data collection tool and procedure

The data collection questionnaire was prepared after reviewing different related literatures. The tools was first prepared in the English language, then translated to Amharic and translated back to English by another translator to check the consistence of the data. A pretested structured questionnaire was used to collect the required data from respondents. Data were collected by face-to-face interview and supervision was based on prepared checklists. Five health extension workers were involved in the data collection and two nurses were assigned as supervisors.

### Data quality assurance

The quality of data was assured by proper designing and pretest of the questionnaires in 5 % (37 mothers) of the sample size in one kebele, other than the selected kebeles, with similar sociodemographic characteristics. Two days training was given for both data collectors and supervisors by the principal investigator before and after the pretest. The training was about the objectives of the study, contents of the questionnaire, data collection techniques and the issues of the confidentiality of the responses. Every day after data collection, the questionnaires were reviewed and checked for completeness by the supervisors and principal investigator and the necessary feedback was offered to the data collectors in the next morning.

### Data processing and analysis

All the questionnaires were checked for completeness, coded and entered into EpiData version 3.1 and then exported to SPSS version 20 for data analysis. The descriptive statistics was presented in the form of tables and text using frequencies and numerical summary statistics such as mean and standard deviation. Variables in the bivariate analysis with *p* - value ≤ 0.2 were further considered in the final logistic regression analysis. The degree of association between independent and dependent variables was assessed by using odds ratio at 95 % confidence interval.

## Results

### Socio- demographic characteristics

Seven hundred thirty nine mothers who had children less than 2 years of age were included in the study making the response rate of 100 %. Out of the total children, 400 (54.1 %) and 339 (45.9 %) of the children were males and female respectively. The mean (± SD) age of the mother and the child were 31 (±6) years and 12 (±7) months respectively. Out of the total study participants, 475 (64.3 %) of mothers earn an average monthly income of less than 500birr, 336 (45.5 %) mothers were unable to read and write and only 453 (61.3 %) mothers have access to Media. The majority, 711 (96.2 %) of respondents were married (Table 1).

**Table 1:** Socio-demographic characteristics of mothers who had a child less than 2 years of age in Dembecha Zuria Woreda, North West Ethiopia, 2015

Variables (*n* = 739)	Frequency	Percent
Maternal age		
15–19 year old	15	2
20–24	84	11.4
25–29	223	30.2
30 +	417	56.4
Child age in months		
< 6 months	203	27.5
≥ 6 months	536	72.5
Religion of the mother		
Orthodox	735	99.5
Muslim	4	0.5
Maternal education		
Unable to read and write	336	45.5
Read and write	332	44.9
Elementary (grade 1–8)	48	6.5
High school (grade 9–12)	19	2.6
College level and above	4	0.5
Marital status		
Married	711	96.2
Single	13	1.8
Divorced	12	1.6
Widowed	3	0.4
Occupation of the mother		
Housewife	682	92.3
Farmer	12	1.6
Private work	4	0.5
Daily laborer	40	5.4
Others	1	0.1
Husband’s education		
Unable read and write	232	31.4
Read and write	431	58.3
Elementary (1–8)	46	6.2
High school (9–12)	17	2.3
College and above	13	1.8
Monthly income (in Ethiopian birr)		
< 500.00 birr	475	64.3
501.00–1499.00 birr	181	24.5
> 1500.00 birr	83	11.2
Media access (radio/television)		
Yes	453	61.3
No	286	38.7

### Health service and obstetrics related history

Most, 710 (96.1 %) of the mothers attended an antenatal visit during their last pregnancy and 514 (69.6 %) of the mothers have history of three and above pregnancies. Eighty three percent of mothers gave birth to their previous baby at the health institution. More than 2/3 of mothers had received postnatal care after their last delivery and 432 (58.5 %) of mothers received information about breastfeeding during antenatal visits (Table 2).

**Table 2:** Obstetric history of mothers who had a child under 2 years of age in Dembecha Zuria Woreda, North West Ethiopia, 2015

Variables (*n* = 739)	Frequency	Percent
Gravidity (*n* = 739)		
One	100	13.5
Two	125	16.9
Three & above	514	69.6
Antenatal visit in the last pregnancy (*n* = 739)		
Yes	710	96.1
No	29	3.9
Number of antenatal visits in the last pregnancy		
One	22	3.00
Two-three	275	37.2
Four & above	413	55.9
Breastfeeding counseling during antenatal appointments (*n* = 739)		
Yes	432	58.5
No	278	37.6
Place of delivery (*n* = 739)		
Health facility	612	82.8
Home	127	17.2
Attended at birth		
Traditional birth attendant	121	16.4
Health professionals	618	83.6
Postnatal visit (*n* = 739)		
Yes	562	76.0
No	177	24.0
Breastfeeding counseling during postnatal visit (*n* = 739)		
Yes	556	75.2
No	7	0.9
History of infant admission (*n* = 739)		
Yes	59	8.0
No	680	92.0
History of breast problems (*n* = 739)		
Yes	62	8.4
No	677	96.1

### Knowledge and attitude towards timely breastfeeding initiation

The majority, 552 (74.7 %) of the respondents have good knowledge, while 187 (25.6 %) had poor knowledge about the timely breastfeeding initiation. The majority, 661 (89.4 %) of the respondents have a favourable attitude and 78 (10.6 %) have unfavourable attitude towards the timely initiation of breastfeeding.

### Breastfeeding practice

The level of timely initiation of breastfeeding was 540 (73.1 %) and the majority, 725 (98.1 %) of mothers ever breastfed. More than two thirds, 563 (76.2 %) of mothers fed their colostrum, 87 (11.8 %) of the mothers were giving the child foods other than breast milk in the first six months after delivery; 61 (70.1 %) mothers gave water, 17 (2.3 %) mothers gave butter, 4 (0.5 %) gave glucose water and 5 (0.7 %) mothers gave other food. The majority, 569 (77 %) of mothers have received breastfeeding advice and the majority, 533 (72.1 %) of mothers were advised by health professionals. Two hundred and three mother’s breastfed their child when the child cries and 736 (99.6 %) mothers were still breastfeeding their child (Table 3).

**Table 3:** Breastfeeding practice of mothers who had a child less than 2 years of age in Dembecha Zuria Woreda, North West Ethiopia, 2015

Variables (*n* = 739)	Frequency	Percent
Did you breastfeed your last child?		
Yes	725	98.1
No	14	1.9
Did you breastfeed within one hour?		
Yes	540	73.1
No	199	26.9
Did you feed your colostrum to your last child?		
Yes	563	76.2
No	176	23.8
Did you use prelacteal foods with your last child?		
Yes	87	11.8
No	652	88.2
If yes, what types of prelacteal food did you use?		
Butter	17	2.3
Water	61	8.3
Glucose water	4	0.5
Others	5	0.7
When do you breastfeed this last child?		
On demand	457	61.8
When the child cries	273	36.9
Other	9	1.2
Do you still breastfeed?		
Yes	736	99.6
No	3	0.4
Any breastfeeding difficulty with your last child?		
Yes	115	15.6
No	624	84.4
Breastfeeding frequency of your last child		
Every ≤ 2 h	383	51.8
Every > 2 h	356	48.2
Did you receive counseling about breastfeeding with your last child?		
Yes	569	77
No	170	23
Who advised you about breastfeeding?		
Health professional	533	72.1
Friend	23	3.1
Family	13	1.8

### Factors associated with timely initiation of breastfeeding

Bivariate and multivariate analyses were conducted. On bivariate analysis, factors which were associated with timely initiation of breastfeeding include; Media access (like radio and/or television), number of antenatal visits during the last pregnancy, place of delivery of the last child, attitude of respondents towards timely breastfeeding initiation, and traditional birth attendance. Candidate variables for multivariate analysis were entered to multiple logistic regression analysis and variables which were found to be associated with timely breastfeeding initiation include; the presence of four and above antenatal visits during the last pregnancy (AOR 3.1, 95 % CI 1.2, 8), access to mass media (AOR 1.54, 95 % CI 1.10, 2.20), and presence of a traditional birth attendant during childbirth (AOR 0.23, 95 % CI 0.07, 0.75) (Table 4).

**Table 4:** Multivariable logistic regression analysis showing factors associated with timely initiation of breastfeeding among mothers who had a child less than 2 years of age in Dembecha Zuria Woreda, North West Ethiopia, 2015

Variables		Timely initiation of breastfeeding		COR (95 % CI)	AOR (95 % CI)	*p* -value
		Within 1 h of childbirth n (%)	After 1 h of childbirth n (%)			
Number of antenatal visits	1 visit	9 (40.9 %)	13 (59.1 %)	1	1	
	2–3	180 (65.5 %)	95 (34.5 %)	2.74 (1.13, 6.64)*	1.74 (0.68, 4.42)	0.248
	≥4	330 (79.9 %)	83 (20.1 %)	5.74 (2.37, 13.89)*	3.1 (1.2, 8)	0.019*
Access to mass Media (e.g.: radio, television)	Yes	349 (77 %)	104 (23 %)	0.59 (0.43, 0.93)*	1.54 (1.1, 2.2)	0.018*
	No	191 (66.8 %)	95 (33.2 %)	1	1	
Place of delivery of the last child	Health facility	470 (76.8 %)	142 (23.2 %)	2.7 (1.8, 4.01)*	1.84 (0.564, 5.988)	0.312
	Home	70 (55.1 %)	57 (44.9 %)	1	1	
Attitude towards timely initiation of breastfeeding	Unfavorable attitude	373 (75.7 %)	120 (24.3 %)	1	1	
	Favorable attitude	167 (67.9 %)	79 (32.1 %)	0.68 (0.49, 0.95)*	1.34 (0.93, 1.92)	0.118
Last child birth attended by	Skilled birth attendant	62 (51.2 %)	59 (48.8 %)	1	1	
	Traditional birth attendant	478 (77.3 %)	140 (22.7 %)	3.25 (2.17, 4.86)*	0.23 (0.07, 0.75)	0.014*

*AOR* adjusted odd ratio, *COR* crude odd ratio

*Statistical significance at (*p* - value < 0.05), 1: reference group

## Discussion

The findings of this study showed that the overall level of timely initiation breastfeeding of mothers who had less than 2 years of age were 540 (73.1 %). This was much lower than the Ethiopian Ministry of Health target to increase breastfeeding within the first hour of life to 92 % by 2015 [8]. The finding is also lower that a study conducted in Bahir Dar town (87.0 %) [13], and Australia 98 % [14] and slightly lower than Saudi Arabia (77.8 %) [15]. The finding was slightly higher than those observed in other studies from South Nation Nationality of People (67 %), Dire Dawa (66 %), and Nepal (66.4 %) [8, 16–21]. This difference may be due to health policy difference among the countries and due to the difference in sociodemographic characteristics. In addition, the current study was conducted in a relatively rural area when compared with the above mentioned studies. This finding is also similar with a study conducted in Goba town [9].

Only 76.2 % of women reported that they feed their colostrum to their child, while 23.8 % discarded their colostrum before breastfeeding. The finding of this study is lower than a study in Axum, in which only 55 % didn’t express and discard their colostrum [20]. The possible explanations for such difference could be due to the difference in sociodemographic and study period; the current study was conducted after large scale educational programs on the importance of feeding colostrum during the recent years.

The magnitude of prelacteal feeding in this study was 11.9 %. Compared to other studies, this finding is almost similar with a study done in Axum, in which the prevalence of prelacteal feeding was 11.72 % [20]. The finding is relatively lower than the study done in India, 16.9 % [6].

The multivariate logistic regression model found three factors associated with timely initiation of breastfeeding. These variables include; the presence of four and above antenatal appointments during the last pregnancy, access to mass media (e.g. radio, television), and traditional birth attendance during their last birth.

Mothers who had four and above antenatal care visits during their previous pregnancy were three times more likely to initiate breastfeeding within one hour after childbirth (AOR 3.1, 95 % CI 1.2, 8). This could be because mothers who had frequent antenatal care visits during their pregnancy could access frequent counselling sessions on the importance of timely initiation of breastfeeding, and thereby be more likely to practice it. This finding is supported by a study done in western Ethiopia and in India, which showed that counselling on breastfeeding during antenatal visits increases the rate of timely initiation of breastfeeding by mothers [1, 6, 22]. This is because the mother becomes receptive and prepares herself for timely breastfeeding [6]. The findings are also similar with the findings of large scale community based programs as conducted in Bolivia and Madagascar [23].

Mothers who had access to mass medias like radio, and/or television were more than 1.5 times more likely to initiate breastfeeding within one hour after childbirth (AOR 1.54, 95 % CI 1.1, 2.2). This maybe because the information obtained from such channels regarding breastfeeding improves their knowledge and practice.

Several studies have showed that the use of a skilled birth attendant during childbirth is important in reducing the high level of child and maternal morbidity and mortality especially in developing countries [24–26]. The current study also found that women who were attended by a traditional birth attendant during their last childbirth were less likely to start breastfeeding within one hour after birth (AOR 0.23, 95 % CI 0.07, 0.75). The possible explanation for this could be difference in the health education provided by health professionals as part of labour and delivery care, and immediately after child birth when compared with traditional birth attendants.

The study had certain limitations. The limited nature of the cross-sectional study design in determining cause-effect relationship, and recall bias may be introduced since this study also included mothers who experienced childbirth before the 2 years of the data collection period.

## Conclusions

Although some improvements have been observed, the study showed that the level of timely initiation of breastfeeding is still low. Even though a large number of women are feeding their babies their colostrum, a significant is the number of women are still discarding their colostrum before initiating breastfeeding. Timely initiation of breastfeeding was positively associated with the presence of four and above antenatal care visits during the previous pregnancy, and access to mass media (e.g. radio, television). In addition, women whose last child was attended by a traditional birth attendant were less likely to initiate breastfeeding within the first one hour after birth. Community based programs focusing on improving knowledge of mothers towards timely breastfeeding initiation is important. Policy makers should give emphasis on the factors mentioned above when designing interventions to improve the practice on timely breastfeeding initiation. Further studies should be conducted on a larger scale for reasons of not feeding colostrum.

## Abbreviations

AOR: Adjusted odd ratio
COR: Crude odd ratio
FMOH: Federal Ministry of Health
TBA: Traditional birth attendant
WHO: World Health Organization
